# Failure Modes and Survival of Anterior Crowns Supported by Narrow Implant Systems

**DOI:** 10.1155/2020/1057846

**Published:** 2020-09-07

**Authors:** Edmara T. P. Bergamo, Everardo N. S. de Araújo-Júnior, Adolfo C. O. Lopes, Paulo G. Coelho, Abbas Zahoui, Ernesto B. Benalcázar Jalkh, Estevam A. Bonfante

**Affiliations:** ^1^Department of Prosthodontics and Periodontology, Bauru School of Dentistry-University of Sao Paulo, Bauru 17012-980, Brazil; ^2^Department of Biomaterials and Biomimetics, New York University College of Dentistry, USA; ^3^Department of Biomedical Engineering, New York University Tandon School of Engineering Brooklyn, USA; ^4^Hansjörg Wyss Department of Plastic Surgery, New York University Grossman School of Medicine, New York City, 10010 NY, USA

## Abstract

The reduced hardware design of narrow implants increases the risk of fracture not only of the implant itself but also of the prosthetic constituents. Hence, the current study is aimed at estimating the probability of survival of anterior crowns supported by different narrow implant systems. Three different narrow implant systems of internal conical connections were evaluated (Ø3.5 × 10 mm): (i) Active (Nobel Biocare), (ii) Epikut (S.I.N. Implant System), and (iii) BLX (Straumann). Abutments were torqued to the implants, and standardized maxillary incisor crowns were cemented. The assemblies were subjected to step-stress accelerated life testing (SSALT) in water through load application of 30 degrees off-axis lingually at the incisal edge of the crowns using a flat tungsten carbide indenter until fracture or suspension. The use level probability Weibull curves and reliability for completion of a mission of 100,000 cycles at 80 N and 120 N were calculated and plotted. Weibull modulus and characteristic strength were also calculated and plotted. Fractured samples were analyzed in a stereomicroscope. The beta (*β*) values were 1.6 (0.9-3.1) and 1.4 (0.9-2.2) for BLX and Active implants, respectively, and 0.5 (0.3-0.8) for the Epikut implant, indicating that failures were mainly associated with fatigue damage accumulation in the formers, but more likely associated with material strength in the latter. All narrow implant systems showed high probability of survival (≥95%, CI: 85-100%) at 80 and 120 N, without significant difference between them. Weibull modulus ranged from 6 to 14. The characteristic strength of Active, Epikut, and BLX was 271 (260-282) N, 216 (205-228) N, and 275 (264-285) N, respectively. The failure mode predominantly involved abutment and/or abutment screw fracture, whereas no narrow implant was fractured. Therefore, all narrow implant systems exhibited a high probability of survival for anterior physiologic masticatory forces, and failures were restricted to abutment and abutment screw.

## 1. Introduction

Endosseous implants are common therapeutic approaches in oral rehabilitation that support the reconstruction of damaged tissues due to trauma/pathology by employing implant-supported prosthetic devices [1, 2], restoring patients' quality of life through natural-like esthetic appearance and masticatory function [3, 4]. Osseointegrated implants have been indicated for over 50 years to rehabilitate from single to full-arch edentulism with high implant survival rates, approximately 95%, and stability of soft and hard peri-implant tissue, and marginal bone loss of approximately 0.50 mm, after 10 years of follow-up [5–7]. Current implant-supported reconstructions have been centered on the use of metal-ceramic, polymeric, and all-ceramic prostheses screwed and/or cemented to prefabricated metallic abutments, with approximately 90% survival rates up to 10 years in function [5, 8–11]. Such increasingly convincing clinical data and bioengineering improvements have encouraged the indication of dental implants in more challenging clinical conditions than originally planned [12, 13], such as in the maxilla where there is less cortical bone to provide initial stability [14, 15]. Similarly, implant loading has been indicated steadily earlier with similar success rates to delayed loading, higher than 90% [16, 17]. Although high survival rates are reported for immediate loading, the indication of this protocol has shown to become less predictive when implant fixtures are placed in critical clinical scenarios, including postextraction sites and nonsplinted single crowns [18].

The interplay between implant macrogeometry and surgical instrumentation, bone availability, and quality have a profound influence on the achievement of optimal primary stability, favoring an undisturbed peri-implant healing, which can render the system a temporal load-bearing capability [19–23]. The characteristics of the implant design, especially the body and apex shape and thread profile, regulate bone response during implant placement, controlling the stress distribution to the surrounding bone and implant stabilization [23–26]. Therefore, implant geometry has significantly evolved over the years to maximize the biomechanical performance, especially in compromised bone scenarios [19–21].

Irrespective of implant design, an optimal three-dimensional implant positioning has to be assured to achieve long-term success, avoiding functional, biological, and esthetic complications [27, 28]. Clinical scenarios of anatomic paucity of the bone (alveolar crest atrophy) and/or compromised osteotomy walls resulting from tooth extractions, where the limited bone availability compromises the use of standard-diameter implants (*θ* ≥ 3.75 mm to *θ* < 5.0 mm) [29], often require bone grafting procedures prior to implant surgery, which prolong treatment time and increase costs, increase morbidity, and frequently compromise immediate or early implant loading [30, 31]. Therefore, alternative concepts such as the use of narrow diameter implants (*θ* < 3.75 to *θ* ≥ 3.0 mm) have raised as potential clinical options to rehabilitate areas with limited prosthetic space [29], with approximately 10% reduction in the need for bone tissue manipulation and respecting the minimum requirements for adequate papillary fill [27, 28, 31–35]. Narrow implants have shown similar survival rates to standard diameter implants, higher than 95%, and marginal bone loss of approximately 2 mm after an average 4 years of follow-up [36–38]. Moreover, reduced diameter implants have been successfully indicated in immediate loading protocols, with no implant loss and approximately 0.2 mm marginal bone loss after 2 years, which requires further long-term investigations [39].

Despite the high survival rates, caution has been advised in the use of narrow implant systems, where not only the implant itself but also the prosthetic constituents might be more prone to fatigue damage accumulation and fracture as a result of their reduced hardware design [40–43]. Moreover, the smaller stress distribution area of narrow implants may have a major impact on the ability to withstand biting forces, leading to bone overloading [40]. To the authors' knowledge, there is currently no study evaluating the biomechanical performance of newly developed implant systems, comprising of a narrower conical body shape and reduced neck diameter. Considering that complex mechanical loading scenarios play a significant role in the strength degradation of implant systems in the oral environment, a laboratory fatigue testing that reproduces clinical failures, such as step-stress accelerated life testing (SSALT), becomes an important tool to predict the lifetime of the implant-abutment-prosthesis reconstructions [42, 44–47]. Hence, the present study used SSALT to estimate the probability of survival and failure mode of the recently developed narrow implant systems. The postulated null hypothesis was that different narrow implant systems would not result in different probability of survival.

## 2. Materials and Methods

### 2.1. Sample Preparation

**Figure 1:**
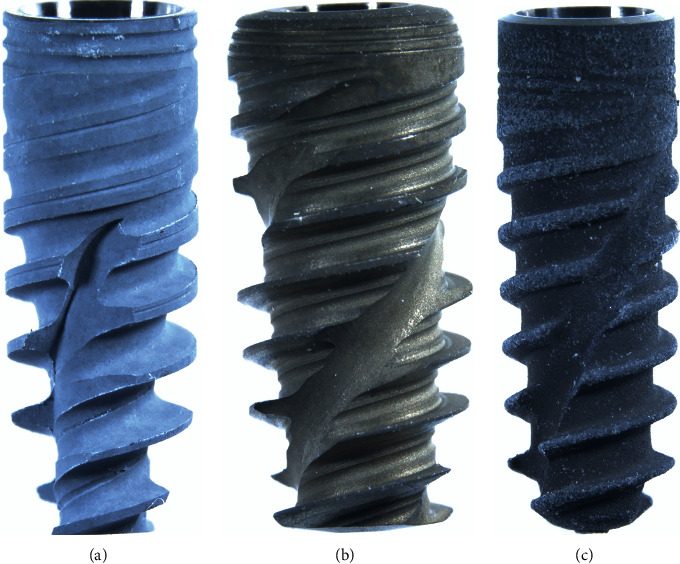
Representative images of the implants' macrogeometry: Active (a), Epikut (b), and BLX (c).

Three narrow implant systems of internal conical connections were evaluated in the current study (Ø3.5 × 10 mm/*n* = 21/implant system): (i) Active (Nobel BioCare, Zürich, Switzerland), (ii) Epikut (S.I.N Implants, Sao Paulo, SP, Brazil), and (iii) BLX (Straumann, Basel, Switzerland). Such implant systems consisted of a narrower conical body shape with a reduced neck diameter as well as a large thread pitch, deep and widen threads, with the ability to cut the bone (the sharpness varies according to the length of the implant) (Figure 1).

Sixty-three implants were fixed in a surveyor (B2, Bio-ART, Sao Carlos, SP, Brazil) to standardize the position and embedded using polymethylmethacrylate acrylic resin (Orthodontic Resin, Dentsply, York, PA, USA) into a 15 mm diameter matrix at the same level of the implant platform. Proprietary Ti-base abutments (Pillar Snappy, Nobel; Duotech, S.I.N. Implant System; Variobase, Straumann) were torqued to the implants' respective groups, using a digital torque gauge (Tohnichi BTG150CN-S, Tohnichi America, Buffalo Grove, IL, USA), following the manufacturer's instruction.

Standardized maxillary central incisor crowns were virtually designed; the wax was pattern milled and casted using cobalt-chrome alloy (Wirobond 280, BEGO, Lincoln, RI, USA). The crowns were cemented on the abutments using a self-adhesive dual-curing resin cement (Rely X U200, 3 M Oral Care, St. Paul, MN, USA), following manufacturer's instructions.

### 2.2. Fatigue Testing

Single load-to-failure (SLF) testing was performed in three specimens of each group to design the stress profiles for the step-stress accelerated life testing (SSALT). An uniaxial compression load was applied 30 degrees off-axis lingually at the incisal edge of the crown using a flat tungsten carbide indenter at a crosshead speed of 1 mm/min (ElectroPuls™ E3000 Linear-Torsion System, Instron, Norwood, MA, USA) [41, 45, 47, 48]. The remaining eighteen specimens per implant system were assigned to the three stress profiles following the ratio distribution of 3 : 2 : 1, where 9 were allocated in the mild, 6 in the moderate, and 3 in the aggressive, as detailed elsewhere [41, 45, 47, 48]. These profiles are named based on the load increase rapidness, in which a specimen will be fatigued throughout the cycles until a certain load level. It means that specimens allocated in the mild profile will be cycled for a longer time to reach the same load level of a specimen assigned to the moderate or aggressive profiles.

SSALT was performed using the same all-electric dynamic test equipment, where the load was also applied 30 degrees off-axis lingually at the incisal edge of the crown using the same flat tungsten carbide indenter at a frequency of 15 Hz in water until specimen failure (considered a fracture or bending of the abutment, abutment screw, or implant) or survival (no failure at the end of the step-stress profiles when testing was suspended), until a maximum load of 500 N. The findings were recorded as stress profile, load at failure, and number of cycles.

Based on the failure distribution, the data was analyzed using an underlying life distribution to describe the life data collected at different stress levels and a life-stress relationship to quantify the manner in which the life distribution changed across different stress levels [45, 49–51]. Thus, the Weibull Distribution was chosen to fit the life data collected in SSALT and its probability density functions (pdfs) was given by (Equation (1)):

(1) $$\begin{array}{c} \int \left(T\right)=\frac{\beta }{\eta \;}\;{\left(\frac{T}{\eta }\right)}^{\beta -1}{ℯ}^{{\left(T/\eta \right)}^{\beta }}, \end{array}$$

where *η* is the scale parameter and *β* is the shape parameter. Considering the time-varying stress model (*x*(*t*)), the inverse power law relationship (IPL) was selected to extrapolate a use level condition considering the cumulative effect of the applied stresses, commonly referred as the cumulative damage model. In such a model, the IPL would be given by (Equation (2)):

(2) $$\begin{array}{c} \;L\left(x\left(t\right)\right)={\frac{\alpha }{x\left(t\right)}}^{\eta }, \end{array}$$

where *L* is the life data and *x*(*t*) is the stress. Then, the IPL-Weibull pdf (where *η* is replaced by the IPL) was given by (Equation (3)):

(3) $$\begin{array}{c} \int \left(t,x\left(t\right)\right)=\beta {\left(\frac{x\left(t\right)}{\alpha }\right)}^{n}{\left(\int_{0}^{t}{\left(\frac{x\left(t\right)}{\alpha }\right)}^{n}du\right)}^{\beta -1}e^{-{\left({\left(\left(x\left(t\right)\right)/\alpha \right)}^{n}du\right)}^{\beta }}. \end{array}$$

From the extrapolated use level pdf, a variety of functions was derived, including reliability (Equation (4)):

(4) $$\begin{array}{c} R\left(t,x\left(t\right)\right)=e^{-{\left({\left(\left(x\left(t\right)\right)/\alpha \right)}^{n}du\right)}^{\beta }}. \end{array}$$

Parameter estimation for all analyses was calculated via MLE method, and 90% two-sided confidence interval (90% CI) was approximated using the Fisher matrix approach. Hence, the use level probability Weibull curves (probability of failure versus number of cycles) with a set load of 100 N were calculated and plotted (Synthesis 9, Alta Pro, Reliasoft, Tucson, AZ, USA). The reliability was calculated for the completion of a mission of 100,000 cycles at 80 and 120 N, and the differences between groups were identified based on the nonoverlap of the CI. The use level probability Weibull analysis provides the beta (*β*) value, which describes the failure rate behavior over time (*β* < 1 indicates that failure rate decreased over time, *β*~1 failure rate does not vary over time, and *β* > 1 means that failure rate increased over time) [45]. As the calculated use level probability Weibull *β* parameter of the Epikut group was <1, a Weibull 2-parameter calculation of the Weibull modulus, a unitless parameter that measures the variability of the results and the characteristic strength, load at which 63.2% of the specimens would fail, was presented using the final load to failure or survival (Weibull 9++, Reliasoft) [45, 50, 51]. Weibull 2-parameter contour plot (Weibull modulus vs. characteristic strength) was graphed to determine statistical differences through the nonoverlap of CI.

All failed specimens were evaluated in a polarized light stereomicroscope (AxioZoom V16, Zeiss, Oberkochen, Germany) using Z-stack mode which automates sequential imaging along the z-plane and sticks them within the same depth of focus (ZEN 2.3 PRO, Zeiss) to depict fracture planes and allow fractographic analysis under higher magnifications (up to 260x) and classified according to the failure criteria.

## 3. Results

**Figure 2:**
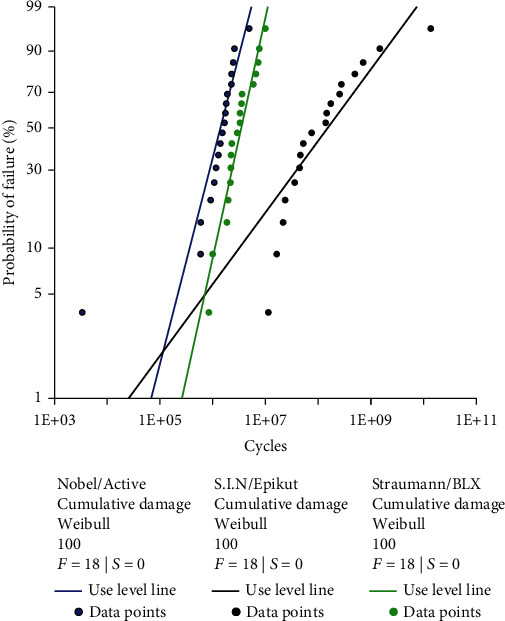
Use level probability Weibull curves at a set load of 100 N showing the probability of failure (%) as a function of cycles of the different narrow implant systems.

All specimens failed during step-stress accelerated life testing (SSALT) testing. The use level probability Weibull curves calculated from the SSALT data for a use level load of 100 N are plotted in Figure 2. The mean beta (*β*) values derived from use level probability Weibull calculation were *β* = 1.6 (0.9-3.1) and *β* = 1.4 (0.9-2.2) for the BLX and Active implants, indicating that failures were mainly dictated by fatigue damage accumulation and tended to increase over time, while the lower bound values of the confidence interval also suggest the influence of material strength. In contrast, the Epikut implant presented *β* = 0.5 (0.3-0.8), indicating that failures were most likely dictated by material strength rather than damage accumulation and tended to decrease over time.

The calculated probability of survival with the corresponding 90% confidence intervals for a determined mission of 100,000 at 80 and 120 N is presented in Table 1. All narrow implant systems investigated, Active (99% and 96%), Epikut (99% and 95%), and BLX (100% and 99%), demonstrated high probability of survival for set missions (80 and 120 N, respectively) that represent above human bite forces (14.5 N) [52], without statistically significant difference between them.

There was no statistically significant difference between narrow implant systems for all estimated missions.

**Figure 3:**
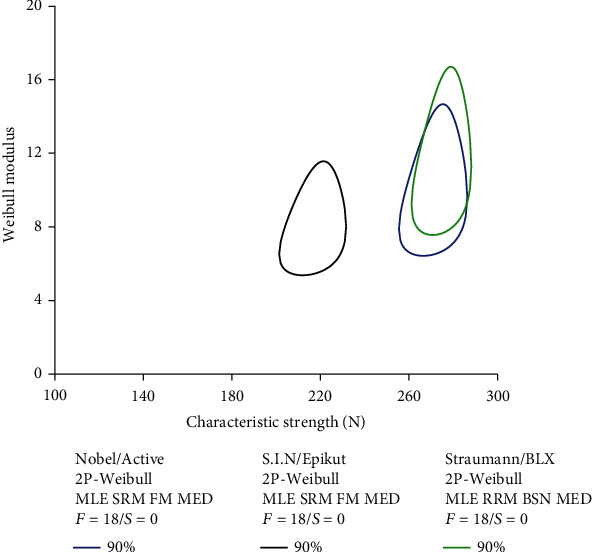
Contour plot showing the Weibull modulus (*m*) as a function of characteristic strength (N). The nonoverlap between contours indicates statistical difference.

The calculated Weibull modulus and characteristic strength are depicted in Figure 3. Active (10, 7.4-13.8), Epikut (8.2, 6.1-10.9), and BLX (11.6, 7.9-14.8) implants exhibited similar Weibull modulus. In contrast, Active (271, 260-282 N) and BLX (275, 264-285 N) implants demonstrated statistically significant higher characteristic strength relative to the Epikut implant (216, 205-228 N); however, all values were higher than the maximum voluntary bite forces reported in the anterior region, approximately 200 N [53].

**Figure 4:**
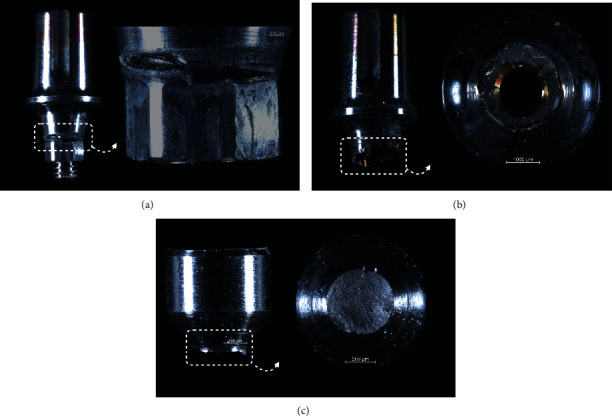
Active group implant failure modes represented by abutment fracture in the area of connection with the implant (a and b) and/or abutment screw fracture (c).

**Figure 5:**
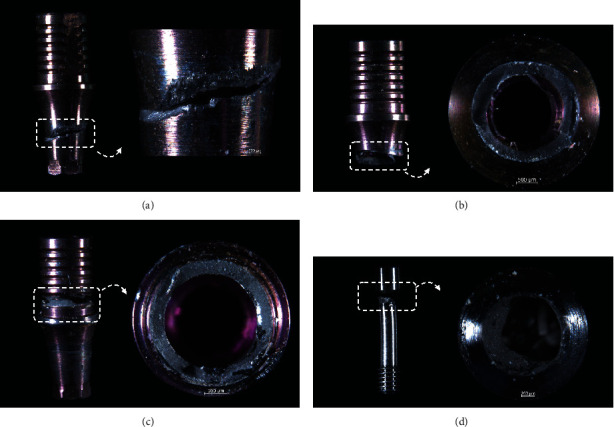
Epikut implant failure modes represented by abutment fracture in the area of connection with the implant (a and b) or the abutment platform where crown is settled (c) and/or abutment screw fracture (d).

**Figure 6:**
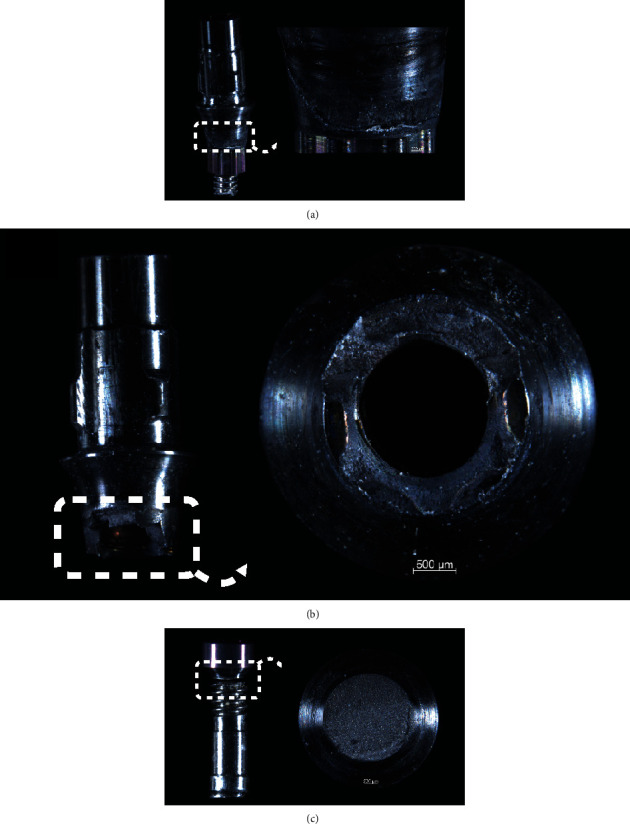
BLX implant failure modes represented by abutment fracture in the area of connection with the implant (a and b) and/or abutment screw fracture (c).

**Figure 7:**
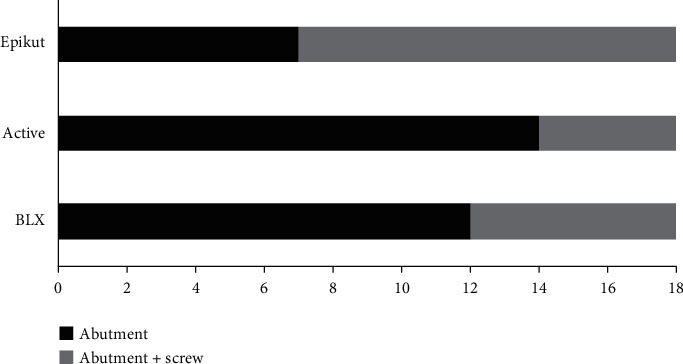
Failure distribution as a function of the narrow implant system.

Representative failed specimens are depicted in Figures 4–6. The failure mode predominantly involved abutment and/or abutment screw fracture, mainly from lingual to buccal where forces physiologically take place, whereas no narrow implant was fractured. While Active and BLX implant fractures were more restricted to the abutment, Epikut fracture predominantly involved abutment, at the connection with the implant and also at the abutment platform where the crown is seated, and abutment screw (Figure 7).

## 4. Discussion

Previous studies have demonstrated that not only narrow implants but also the respective prosthetic components may be more prone to fatigue damage accumulation and fracture due to their smaller hardware design [40–43, 48], which has not yet been investigated for the newly developed implant systems, whose original strategy suggests a narrower bulk design with a more pronounced thread profile. Hence, the current study sought to estimate the fatigue lifetime and probability of survival of anterior crowns supported by recently developed narrow implant systems. From a fatigue perspective, all narrow implant systems showed high probability of survival for determined missions equivalent to anterior physiologic masticatory forces; thus, the postulated null hypothesis that different narrow implant systems would not result in different probability of survival failed to be rejected.

The current biomechanical findings obtained after fatigue testing the narrow implant systems have been related to the combination of degradation mechanisms associated with repeated loading and damage accumulation as well as strength of the weakest component of the implant-supported reconstruction, the abutment and/or abutment screw [45]. While fatigue predominantly accelerated the failure of BLX and Active narrow implant systems, there has also been an evidence of the influence of the material strength based on lower bound values of the confidence interval. In contrast, the failures of the Epikut system were mainly attributed to material strength rather than fatigue damage accumulation. In fact, flaws intrinsic to material processing can cause a meaningful variation in the fracture load from sample to sample, and the Weibull modulus (*m*, the shape parameter of Weibull distribution) is a dimensionless material-specific parameter used as an indicator of strength variation or asymmetric strength distribution a result of flaw population within the material structure. Higher *m* values indicate a more homogeneous flaw size distribution throughout the material, narrower strength scattering, and, consequently, greater structural reliability [54, 55]. The opposite association is expected from lower *m* values. In the current study, all groups present similar Weibull modulus, indicating similar structural reliability, and the values, ranging from 6 to 14, also corroborated with the results of previous studies evaluating different narrow implant systems under similar fatigue methodology, approximately 6 [41, 48].

At a given mission of 80 N and 120 N, all narrow implant systems evidenced high probability of survival, almost 100%. This data suggests that the newly proposed implant design, irrespective of commercial system, can be a reliable option to replace incisors since mean physiologic masticatory forces in these regions vary within the estimated range (14.5 N) [52]. If one considers maximum voluntary bite force values, although BLX and Active implant have shown significantly higher characteristic strength than Epikut, all systems resulted in higher values (>200 N) than the maximum forces reported in the literature for incisors, approximately 200 N [53]. Previous studies investigating the fatigue lifetime of conventional narrow implant systems using SSALT under similar methodology have also demonstrated high probability of survival for similar estimated missions, approximately 99% [41, 48]. The probability of survival of narrow implants has also been compared to standard-diameter implants, with significant differences in the biomechanical behavior being reported only when hexagonal connections were evaluated [48]. This fact endorses the favorable results described in the current study, where the improved stability and stress distribution inherent to the internal conical connection of the different narrow implant systems supported a high survival prediction to the implant and prosthetic constituents, even in the laboratory reproduction of a challenging clinical scenario through off-axis incisal edge loading. Therefore, it can be assumed that the critical determinant of clinical survivability when smaller-diameter implants are taken into consideration should be centered on the proper selection of the implant connection rather than macrogeometry and thread design differences.

In fact, it has been well-established that mechanical complications are increased for external hexagon connection implants as a result of the reduced screw joint stability and resistance to oblique loads [47, 48]. Internal implant connections, such as the internal conical connections, have been suggested to improve the joint strength increasing lateral and rotational stability as a function of a deeper engagement on the implant-abutment interface, shielding the rehabilitation from mechanical overloading during function [56, 57]. Moreover, the increased contact area of the abutment with the implant internal walls, potentially decreasing the micromotion at the interface and deeply distributing the intraoral forces along the implant longitudinal axis, has also shown to protect the implant itself from fracture, even in a narrow diameter design, as observed in the current study where no implant fracture has been observed [41, 44, 58]. In fact, the current results indicated that the implant-supported rehabilitation strength was limited by the abutment and/or abutment screw fracture for all narrow implant systems, where the high stress concentrated at the implant-abutment connection as cycles elapsed and load increased during fatigue exceeded their yield strength, leading to fracture. To overcome the strength limitation of two-piece abutments, the use of monolithic abutments connected to internal conical implants has suggested to provide a potential improved biomechanical performance [41, 59, 60], which warrants further investigation for the novel implant design, especially in a scenario of narrow diameter implants where competing failure modes are likely to occur between the thin implant wall and the bulkier abutment [41]. A noteworthy aspect to be discussed in the narrow implant failure modes of the current study is the presence of abutment platform fracture in the Epikut implant, indicating that the stress concentration exceeded the material strength in such area. Thicker abutment cone walls without compromise final superstructure anatomy may be indicated, and an improved biomechanical behavior could be expected for the prosthetic constituents, which also requires further investigations. Finally, given the positive finding of no implant fractures observed for all groups, it can be assumed that grade IV commercially pure cold worked titanium (Active and Epikut) and titanium-zirconium alloy (BLX, 85%Ti-15%Zr) were equally effective in shifting failures toward prosthetic components, which can be replaced with less morbidity compared to removal of a fractured implant and placement of a new one.

The main challenge in the development of new implant-abutment designs relies not only on the improvement of bone and soft tissue response, hastening osseointegration, but also on reducing and/or eliminating the incidence of biological and mechanical failures in the implant-prosthetic devices when in function; however, such biomechanical innovations require a profound preclinical investigation before their wide indication in the clinical setting to understand the potential complications over time. The step-stress accelerated life testing (SSALT) has been widely used in biomaterial science in order to evaluate the failure behavior of design modifications proposed for implant-supported rehabilitations [41, 42, 44–47]. The results of this type of in vitro study extrapolate clinical failure patterns in a timely way allowing the comparison of the mechanical performance of different systems and/or biomaterials [45]. Thus, the current characterization of the fatigue lifetime and failure modes of the recently developed narrow implant design provided an insight into their biomechanical behavior in a highly demanding anterior reconstruction, where single crowns that are not splinted were subjected to a worst-case loading, challenging the structural integrity of either the prosthesis components or implants; however, caution is also advised in the use of narrow implant systems in particularly challenging scenarios, such as patients with parafunction, since previous studies have demonstrated that narrow implant systems can be more prone to failure relative to standard-diameter implants [42, 43], which may be related to the implant-abutment connection design [44, 47], bulk material [61], and prosthesis fixation mode [62], among others [45], and require further comparisons for the novel systems. Moreover, the mechanical testing was limited to single restorations and such assumptions need to be investigated in posterior restorations, fixed dental prostheses, or full-arch reconstructions, where units are splinted. Future clinical trials are highly recommended to support the indication of such implants and benefit patients through reducing the indication of difficult and costly bone grafting procedures.

## 5. Conclusions

From an accelerated fatigue testing perspective, it can be concluded that

- all narrow implant systems exhibited high probability of survival for anterior physiologic masticatory forces
- the failure mode was similar for all implants, restricted to abutment and abutment screw fracture

## Figures and Tables

**Table 1:** Probability of survival (%) with the corresponding 95% CI for a mission of 100,000 cycles and at 80 and 120 N of the different implant systems.

	Active	Epikut	BLX
Upper bound	100	100	100
Probability of survival (80 N)	99	99	100
Lower bound	95	96	99
Upper bound	99	98	100
Probability of survival (120 N)	96	95	99
Lower bound	87	85	97

## Data Availability

Data will be available upon request.
